# WASH and Tsg101/ALIX-dependent diversion of stress-internalized EGFR from the canonical endocytic pathway

**DOI:** 10.1038/ncomms8324

**Published:** 2015-06-12

**Authors:** Alejandra Tomas, Simon O. Vaughan, Thomas Burgoyne, Alexander Sorkin, John A. Hartley, Daniel Hochhauser, Clare E. Futter

**Affiliations:** 1 Department of Cell Biology, UCL Institute of Ophthalmology, University College London, 11-43 Bath Street, London EC1V 9EL, UK; 2 Department of Cell Biology, University of Pittsburgh School of Medicine, Pittsburgh, Pennsylvania 15261, USA; 3 Cancer Research UK Drug-DNA Interactions Research Group, UCL Cancer Institute, Paul O'Gorman Building, University College London, 72 Huntley Street, London WC1E 6BT, UK; 4 Present address: Section of Cell Biology, Division of Diabetes, Endocrinology and Metabolism, Department of Medicine, Imperial College London, Hammersmith Campus, Du Cane Road, London W12 0NN, UK

## Abstract

Stress exposure triggers ligand-independent EGF receptor (EGFR) endocytosis, but its post-endocytic fate and role in regulating signalling are unclear. We show that the p38 MAP kinase-dependent, EGFR tyrosine kinase (TK)-independent EGFR internalization induced by ultraviolet light C (UVC) or the cancer therapeutic cisplatin, is followed by diversion from the canonical endocytic pathway. Instead of lysosomal degradation or plasma membrane recycling, EGFR accumulates in a subset of LBPA-rich perinuclear multivesicular bodies (MVBs) distinct from those carrying EGF-stimulated EGFR. Stress-internalized EGFR co-segregates with exogenously expressed pre-melanosomal markers OA1 and fibrillar PMEL, following early endosomal sorting by the actin polymerization-promoting WASH complex. Stress-internalized EGFR is retained intracellularly by continued p38 activity in a mechanism involving ubiquitin-independent, ESCRT/ALIX-dependent incorporation onto intraluminal vesicles (ILVs) of MVBs. In contrast to the internalization-independent EGF-stimulated activation, UVC/cisplatin-triggered EGFR activation depends on EGFR internalization and intracellular retention. EGFR signalling from this MVB subpopulation delays apoptosis and might contribute to chemoresistance.

## Abstract (web summary)

Stress induces ligand-independent endocytosis of EGF receptor (EGFR), but its fate and role in signalling are not known. Here Tomas *et al*. show that stress-internalized EGFR accumulates and is retained in distinct multivesicular bodies, and delays the onset of stress-induced apoptosis.

## Graphical abstract

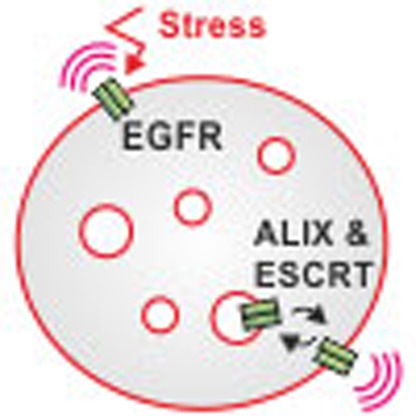

The epidermal growth factor receptor (EGFR) is an archetypal receptor tyrosine kinase (TK) typically activated by ligand binding to its extracellular domain. In the canonical pathway, EGFR phosphorylates downstream targets following activation, initiating signalling cascades that drive various cellular responses, including cell proliferation and inhibition of apoptosis^1^. EGFR is subsequently internalized, and its endocytic trafficking is a major regulatory mechanism for receptor activity, as ligand-induced EGFR lysosomal degradation leads to signal termination^2^. However, the established view that EGFR trafficking functions only for signal termination has been challenged in a number of reports demonstrating that EGFR can continue to signal along the endocytic pathway^3^.

In cancer, EGFR signalling is often deregulated, contributing to tumorigenesis, therapy resistance and poor patient survival^4^. Despite the demonstrated benefits of EGFR-targeting agents, patient responses remain inconsistent and clinical trials in combination with chemotherapy have been disappointing^5^. Although exposure to chemotherapy and other stresses has been shown to elicit ligand-independent EGFR internalization^6^,^7^, the nature of the endocytic compartment(s) through which stress-internalized EGFR traffics, the molecular mechanisms underlying this trafficking and their effects on downstream signalling remain unknown^8^. Here we analyse the post-endocytic fate of the EGFR following exposure to ultraviolet light C (UVC) and the chemotherapeutic drug, cisplatin, and show that EGFR undergoes p38-dependent accumulation in a subpopulation of perinuclear multivesicular bodies (MVBs) that are distinct from those that traffic ligand-stimulated EGFR to the lysosome but contain exogenously expressed markers of melanosome biogenesis. Segregation of stress-induced and ligand-stimulated EGFR into different populations of MVBs depends on the WASP and Scar homologue (WASH) complex that regulates actin dynamics. Unlike ligand-stimulated EGFR, which is activated on the plasma membrane, internalization of stress-induced EGFR is a requirement for its activation. Intracellular retention of stress-induced EGFR in MVBs is independent of EGFR ubiquitination but depends on the endosomal sorting complex required for transport (ESCRT) machinery and ALG-2-interacting Protein X (ALIX) and is required for stress-induced EGFR signalling and protection from apoptosis.

## Results

### Roles of p38 in stress-induced EGFR perinuclear accumulation

**Figure 1:**
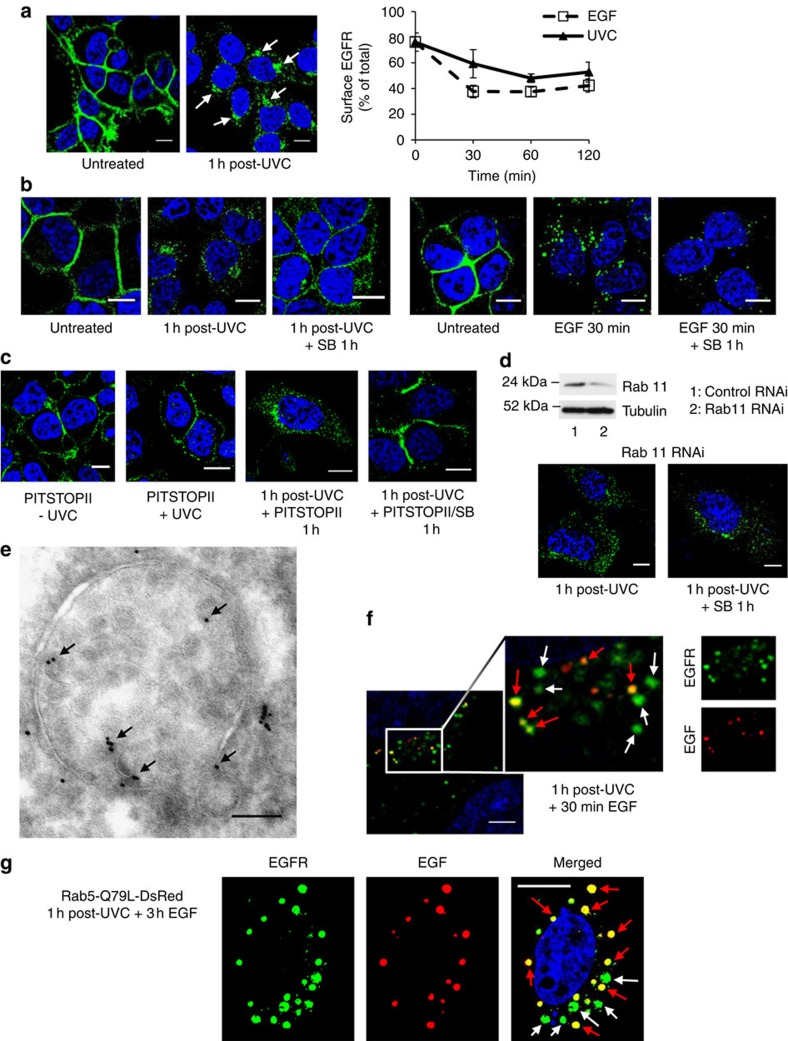
Dual role of p38 in ligand-independent stress-triggered EGFR trafficking in HeLa cells. (**a**) EGFR immunolocalization in untreated versus UVC-exposed cells 1 h post UVC exposure (left), and quantification of surface downregulation in cells exposed to UVC or treated with EGF for the indicated times (right). Arrows indicate perinuclear accumulation of EGFR (green) after UVC exposure. Data are mean±s.e.m. of three independent experiments. (**b**) Untreated or UVC-exposed HeLa cells were fixed after 1 h, or further incubated with the p38 inhibitor SB202190 (SB) for 1 h (left). Untreated or EGF-treated cells were fixed after 30 min, or further incubated with SB for 1 h in the continuous presence of EGF (right). p38 inhibition causes EGFR (green) redistribution to the plasma membrane following UVC, but not EGF exposure. (**c**) Pre-treatment for 30 min with PITSTOPII prevents UVC-induced EGFR (green) internalization (left) but PITSTOPII addition 1 h after UVC-induced EGFR internalization does not affect perinuclear EGFR accumulation or the recycling induced by simultaneous p38 inhibition (right). (**d**) Cells transfected with control or Rab11 siRNA were immunoblotted after 72 h for Rab11 and tubulin to assess knockdown efficiency (top). Rab11 knockdown did not prevent UVC-induced EGFR (green) internalization but prevented EGFR recycling after subsequent SB treatment (bottom). (**e**) Cells transfected with EGFR-GFP were fixed 1 h after UVC exposure and ultrathin cryosections were immuno-labelled for EGFR with 8 nm gold. EGFR-GFP (arrows) is on the limiting membrane and ILVs of MVBs. (**f**) Immunofluorescence analysis of UVC and EGF sequentially exposed HeLa cells (see Methods for experimental details). Red arrows show endosomes containing EGFR (green) and EGF (red). White arrows show EGFR+ve, EGF-ve endosomes, indicating a separate subset of MVBs containing stress-internalized but not EGF-bound EGFR. Scale bar, 5 μm. (**g**) Cells transfected with constitutively active Rab5-Q79L-DsRed were exposed to UVC and incubated for 1 h before treatment with EGF-AlexaFluor 647 (red) for 3 h. Red and white arrows show EGFR+ve/EGF+ve and EGFR+ve/EGF-ve endosomes, respectively. Scale bars, 10 μm for confocal and 100 nm for EM, unless otherwise indicated; 4,6-diamidino-2-phenylindole (DAPI)-stained nuclei, blue.

Immunofluorescence staining of HeLa cells showed that both UVC and cisplatin elicit a striking perinuclear accumulation of internalized EGFR (Fig. 1a and Supplementary Fig. 1a), but western blotting cell lysates showed that, unlike EGF stimulation, they do not induce detectable EGFR degradation (Supplementary Fig. 1b). Quantitative analysis of EGFR surface downregulation showed that the perinuclear intracellular pool of EGFR is maintained for >2 h and accounts for ∼50% of total EGFR (Fig. 1a). In keeping with previous studies, UVC-elicited p38 activity was required to promote initial EGFR internalization (Supplementary Fig. 1c). Interestingly, p38 inhibition after UVC-induced perinuclear EGFR accumulation resulted in redistribution of perinuclear EGFR to the plasma membrane (Fig. 1b), revealing a novel role for p38 in EGFR retention in the perinuclear compartment distinct from its role in EGFR internalization. This is in marked contrast to the p38-independent post-endocytic fate of ligand-stimulated EGFR, which was degraded. Treatment with the clathrin inhibitor PITSTOPII blocked UVC-induced EGFR internalization but when PITSTOPII was added after UVC-induced EGFR perinuclear accumulation to prevent re-internalization of any recycled receptor, the EGFR was retained intracellularly (Fig. 1c). This indicates that UVC-exposed EGFR does not undergo continuous internalization and recycling. However, if p38 is inhibited after UVC-induced EGFR perinuclear accumulation, EGFR recycles to the plasma membrane in a manner that siRNA-mediated depletion shows is dependent on Rab11 (Fig. 1d). In contrast, endocytosis of Transferrin Receptor (TfR) did not require p38 activity, and TfR recycling was unaffected by UVC exposure, indicating EGFR-specific p38-dependent internalization and retention (Supplementary Fig. 1c,d).

### Segregation of stress-exposed EGFR in an MVB subpopulation

UVC-exposed EGFR localized initially to EEA1-positive early endosomes, as previously reported^6^,^9^, but this localization was progressively lost as the receptor accumulated perinuclearly with negligible co-localization with the lysosomal marker Lamp1 (Supplementary Fig. 2a). The striking perinuclear distribution of UVC/cisplatin-stimulated EGFR, together with the availability of this receptor pool for recycling upon p38 inhibition, suggested that EGFR might be sequestered in recycling endosomes. However, stress-activated EGFR showed very limited co-staining with Rab11, although it passed through Rab11-positive recycling endosomes when recycling was induced by p38 inhibition (Supplementary Fig. 2b). To our surprise, high-resolution cryo-immuno electron microscopy (EM) localization of EGFR 1 h post UVC stress revealed that EGFR specifically accumulated in MVBs morphologically indistinguishable from those that traffic ligand-stimulated EGFR (Fig. 1e and Supplementary Fig. 1e). Sequential stimulation first with UVC (which leaves ∼50% EGFR on the cell surface) and then with fluorescent EGF revealed the presence of separate populations of EGFR-containing MVBs in the same cell (Fig. 1f). Similarly, incubating with anti-EGFR gold to track all populations of EGFR and subsequently stimulating first with UVC and then with EGF conjugated to horseradish peroxidase (HRP), allowed the identification of two populations of EGFR-positive MVBs, either containing or not containing EGF (Supplementary Fig. 1f). The remarkable separation of ligand- and stress-stimulated EGFR in distinct endosomal subpopulations was particularly clear in cells with enlarged endosomes following expression of constitutively active Rab5-Q79L (ref. 10); Fig. 1g).

**Figure 2:**
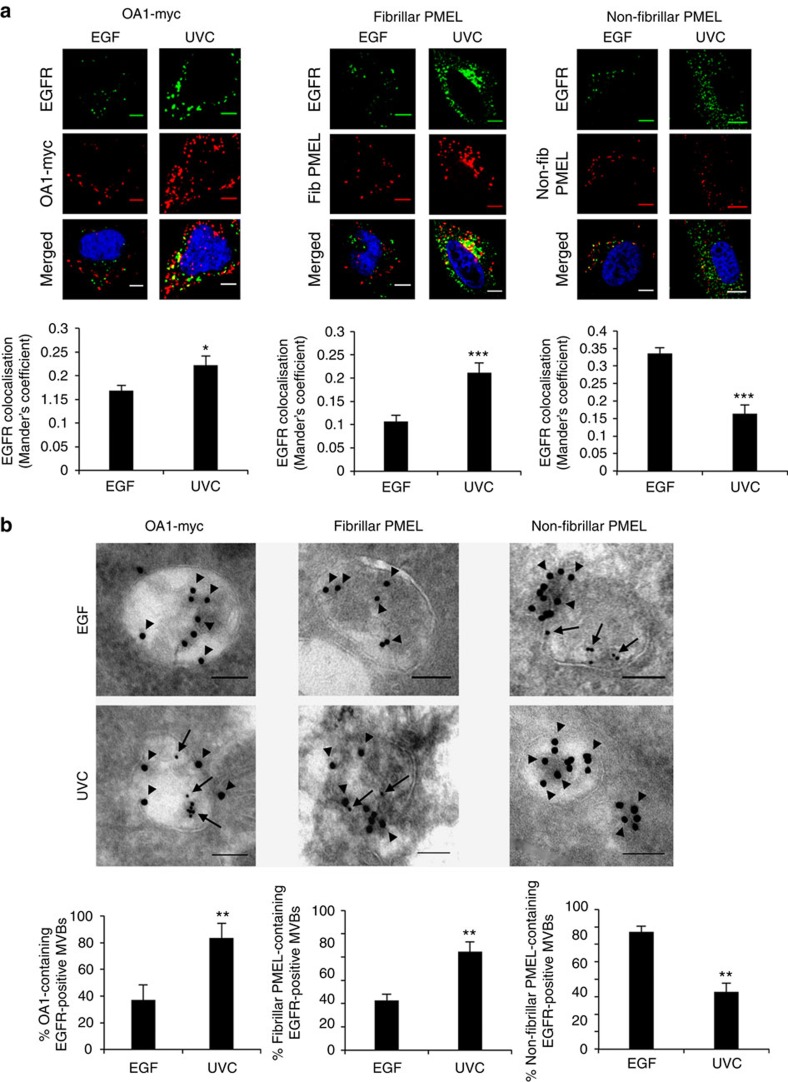
Co-segregation of stress-internalized EGFR with markers of pre-melanosomal MVBs. (**a**) HeLa cells were transfected with OA1-myc or PMEL, and either treated with EGF for 30 min or exposed to UVC and incubated for 1 h. Cells were co-stained with EGFR and either myc (left), fibrillar PMEL (middle) or non-fibrillar PMEL (right). Quantification of co-localization between EGFR (green) and expressed marker (red) for the different conditions is shown below each set of images. Data are mean±s.e.m. of three independent experiments, *P<0.05 and ***P<0.001 (Student's *t*-test). (**b**) HeLa cells were treated as above in the presence of 10 nm anti-EGFR-gold (arrows) before preparation for cryo-immunoEM. Ultrathin cryosections were labelled for myc (left), fibrillar PMEL (middle) or non-fibrillar PMEL (right) with 15 nm-gold (arrowheads). Depicted are typical examples of OA1 and fibrillar PMEL+ve MVBs containing stress-internalized but not EGF-stimulated EGFR, and non-fibrillar PMEL containing EGF-stimulated but not stress-internalized EGFR. Quantification of the percentage of EGFR+ve MVBs containing each of the different markers following EGF versus UVC exposure is shown below each set of images. Data are mean±s.e.m. of ≥10 cells, **P<0.01 (Student's *t*-test). Scale bars, 10 μm for confocal and 100 nm for EM images; 4,6-diamidino-2-phenylindole (DAPI)-stained nuclei, blue.

We have previously shown that ligand-stimulated EGFR is trafficked in a subset of MVBs^11^, but the composition and function of non-EGFR-containing MVBs remain poorly characterized. In this respect, melanosomes are lysosome-related organelles generated from a subset of MVBs diverted from the canonical degradative pathway in pigmented cells^12^. Within those MVBs, premelanosome protein (PMEL) undergoes proteolytic processing to generate fibrils (fibrillar PMEL) upon which melanin is deposited^13^,^14^, whereas unprocessed non-fibrillar PMEL and cleaved C-terminal PMEL fragments containing its transmembrane domain traffic along the degradative pathway^15^. We recently showed that the pre-melanosomal G-protein–coupled receptor OA1, when exogenously expressed in HeLa cells, localizes to a subset of MVBs distinct from those carrying ligand-stimulated EGFR^16^. We now find, by immunofluorescence of HeLa cells transfected with markers of melanosome biogenesis, increased co-localization of EGFR with OA1 and fibrillar PMEL in UVC-exposed, compared with EGF-treated, HeLa cells (Fig. 2a). In contrast, unprocessed non-fibrillar PMEL showed increased co-localization with EGF-bound compared with UVC-exposed EGFR. Parallel cryo-immunoEM showed segregation of stress-exposed EGFR within MVBs containing OA1 and fibrillar PMEL, whereas EGF-bound EGFR segregated within MVBs containing non-fbrillar PMEL and C-terminal PMEL fragments (Fig. 2b and Supplementary Fig. 3a).

### Role of WASH in segregation of stress-exposed EGFR

**Figure 3:**
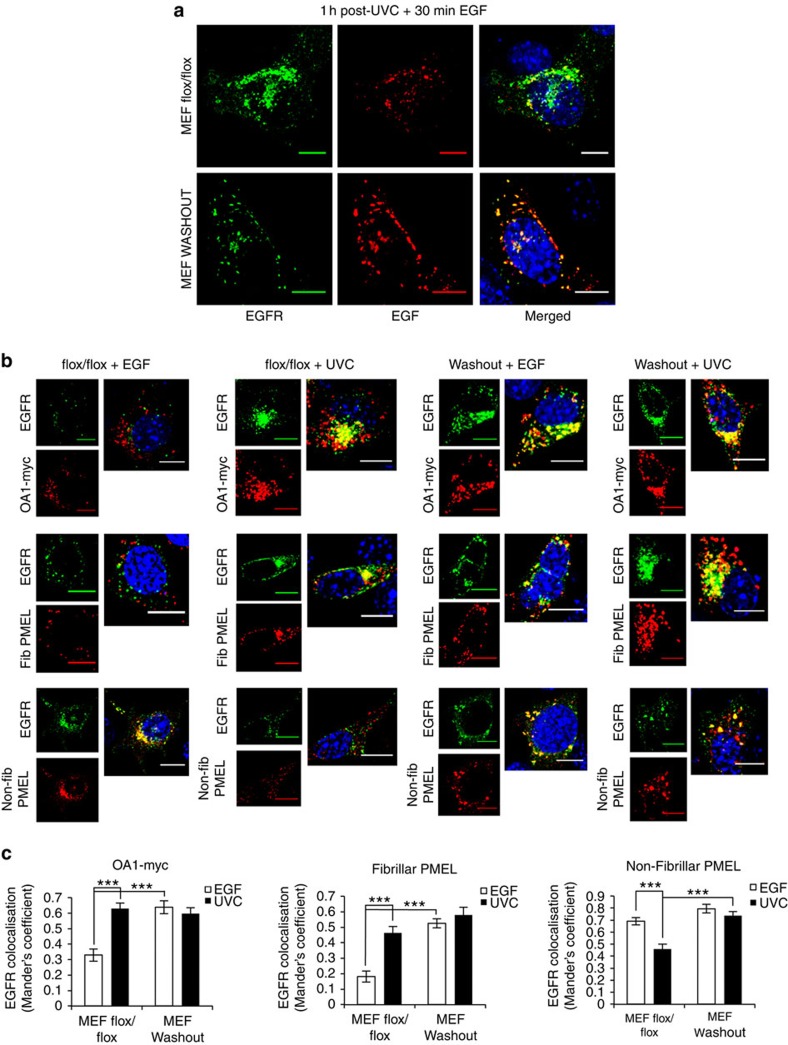
Sorting of EGF-bound and stress-internalized EGFR onto separate MVB subsets is WASH dependent. (**a**) Control flox/flox and WASH knock-out (WASHOUT) MEFs transfected with human EGFR were exposed to UVC and incubated for 1 h to allow stress-induced EGFR internalization in the presence of anti-EGFR 108 antibody. Cells were washed, incubated with EGF-AlexaFluor 488 for 30 min (red), and processed for immunofluorescence with an AlexaFluor 555 secondary antibody to label EGFR (green). Both EGF+ve and EGF-ve EGFR-containing endosomes are present in control flox/flox, but these are largely merged in WASHOUT MEFs. (**b**) Control flox/flox and WASHOUT MEFs were co-transfected with human EGFR and OA1-myc or PMEL, and either treated with EGF for 30 min or exposed to UVC and incubated for 1 h. Cells were co-stained for EGFR (green) and the following markers (in red): myc (top panels), fibrillar PMEL (central panels) or non-fibrillar PMEL (bottom panels). (**c**) Quantification of co-localization between EGFR and expressed markers for the different conditions. Data are mean±s.e.m. of three independent experiments, ***P<0.001 (Student's *t*-test). Scale bars, 10 μm; 4,6-diamidino-2-phenylindole (DAPI)-stained nuclei, blue.

The finding that OA1 and fibrillar PMEL co-segregate with stress-stimulated EGFR provides a means of monitoring the diversion of stress- and ligand-stimulated EGFR from early endosomes. Branched early endosomal actin networks resulting from Arp2/3 activation by the endosomal actin polymerization-promoting complex, WASH^17^, have recently been found to play a vital role in sorting of specific endosomal cargoes to different destinations^18^, such as retromer-mediated endosome-to-Golgi retrieval^19^ and plasma membrane recycling^20^. The WASH complex has also been shown to associate with BLOC-1 (biogenesis of lysosomal organelles complex-1), required for selective cargo exit from early endosomes to melanosomes^21^, suggesting that WASH-mediated actin polymerization may function in melanosome biogenesis^22^. We found that WASH is required for segregation of stress-internalized EGFR away from EGF-bound EGFR by comparing mouse embryonic fibroblasts (MEFs) derived from control flox/flox and Cre-mediated conditional WASH knockout (WASHOUT) embryos^23^. In flox/flox MEFs treated first with UVC and then fluorescent EGF, there are two populations (EGF positive and EGF negative) of EGFR present (Fig. 3a). However, in WASHOUT MEFs, EGFR and EGF signals are totally merged in the same endosomal compartments. Furthermore, although in flox/flox MEFs (like in HeLa cells) UVC-exposed EGFR shows greater co-localization with OA1 and fibrillar PMEL than EGF-bound EGFR, in WASHOUT MEFs UVC-exposed and EGF-bound EGFR co-stain equally well with these markers (Fig. 3b, quantified in Fig. 3c). Similarly, in flox/flox MEFs, EGF-bound EGFR shows greater co-localization with non-fibrillar PMEL than UVC-exposed receptor, but this difference is lost in WASHOUT MEFs. The requirement for the WASH complex in segregation of stress-internalized EGFR along with pre-melanosomal factors away from ligand-stimulated EGFR contrasts with the unimpaired lysosomal degradation of EGF-bound EGFR and plasma membrane recycling of TfR in WASH knockouts^23^.

### Role of ESCRTs/ALIX in MVB retention of stress-exposed EGFR

Following WASH-dependent segregation, stress-stimulated EGFR accumulates in MVBs that do not fuse with lysosomes. To determine whether the failure to fuse with lysosomes was due to stress-induced lysosomal damage, EGFR degradation was measured in cells stimulated first with UVC and then with EGF. UVC-exposed cells clearly retained the capacity for degradation of EGFR (Supplementary Fig. 3b). Furthermore, UVC-exposed cells showed only a minor increase in lysosomal pH, as demonstrated by only marginal reductions in neutral red and lysotracker accumulation (Supplementary Fig. 3c). Taken together, these results indicate that if there is significant lysosomal damage following UVC exposure, this is rapidly recovered and cannot explain the accumulation of EGF in perinuclear MVBs for ≥4 h (Supplementary Fig. 3d).

**Figure 4:**
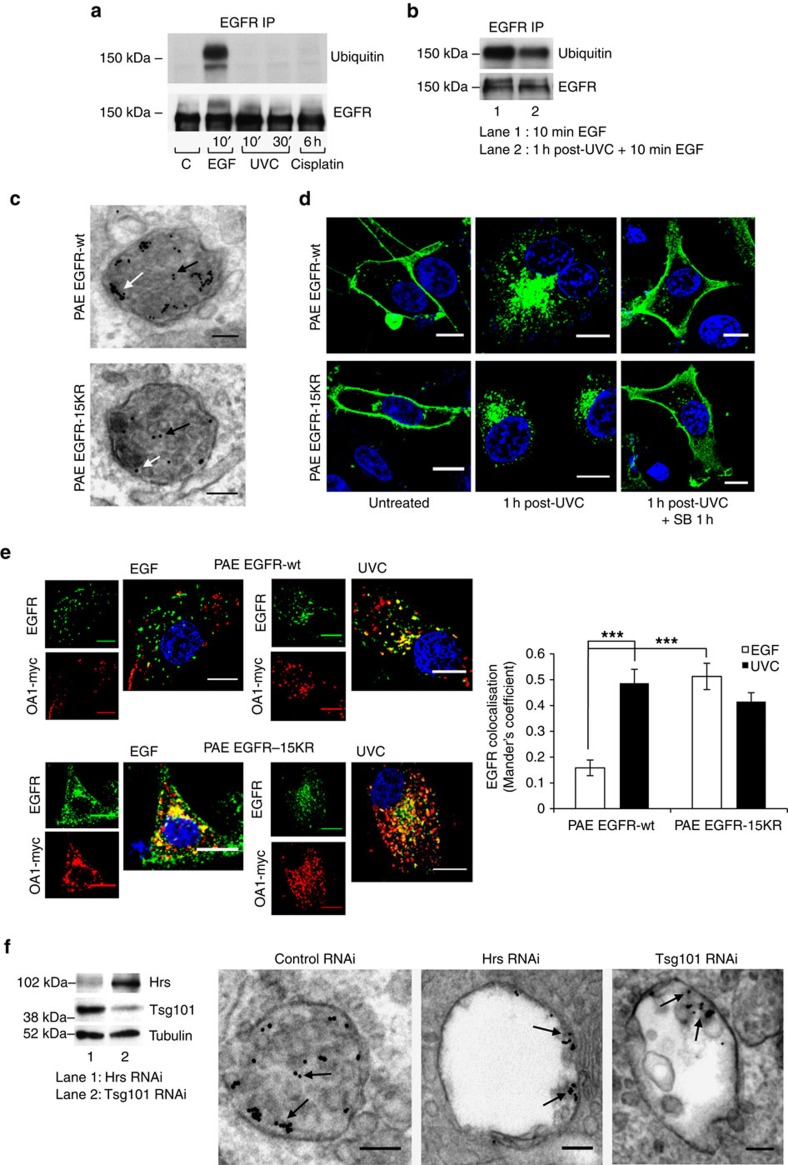
Ubiquitin-independent, ESCRT-dependent sorting of stress-internalized EGFR onto ILVs of MVBs. (**a**) Immunoprecipitation (IP) of EGFR from HeLa cell lysates and immunoblotting for ubiquitin and EGFR showed robust EGFR ubiquitination after EGF stimulation but not after exposure to UVC or cisplatin. (**b**) Treatment of HeLa cells with EGF 1 h post-UVC exposure induced strong EGFR ubiquitination, measured as in **a**, that was reduced compared with EGF alone, most likely because only 50% of EGFR was available for EGF stimulation after UVC exposure. (**c**) Stable PAE cell sublines expressing EGFR-wt or a ubiquitination-defective EGFR (EGFR-15KR) were exposed to UVC and incubated for 1 h with anti-EGFR-gold. Cells were fixed and processed for EM. Representative images of ultrathin sections with gold in ILVs (black arrows) and on the limiting membrane of MVBs (white arrows) from both sublines are shown. (**d**) Immunofluorescence shows perinuclear EGFR (green) accumulation 1 h after UVC exposure followed by recycling to the plasma membrane on subsequent p38 inhibition in both PAE EGFR-wt and -15KR cells, consistent with no role for ubiquitination in stress-induced EGFR traffic. (**e**) PAE EGFR-wt and -15KR cells were transfected with OA1-myc and either treated with EGF for 30 min or exposed to UVC and incubated for 1 h. Ubiquitination-deficient EGFR-15KR (green) showed increased co-staining with OA1-myc (red) following EGF stimulation compared with EGFR-wt, to a similar level to that shown by UVC-internalized EGFR (-wt or -15KR). Data are mean±s.e.m. of three independent experiments, ***P<0.001 (Student's *t*-test). (**f**) Lysates from HeLa cells transfected with Hrs or Tsg101 siRNA were immunoblotted after 72 h for Hrs, Tsg101 and tubulin to assess knockdown efficiency (left). RNAi-treated cells were exposed to UVC, incubated for 1 h with anti-EGFR-gold (arrows) and processed for EM. Ultrathin sections (right) show enlarged MVBs containing reduced numbers of EGFR-positive ILVs in Hrs and Tsg101 siRNA-treated compared with control RNAi cells. Scale bars, 10 μm for confocal and 100 nm for EM images; 4,6-diamidino-2-phenylindole (DAPI)-stained nuclei, blue.

The best characterized MVB sorting machinery is the ESCRT complex system^24^, which recognizes ubiquitinated cargoes such as ligand-stimulated EGFR and sorts them onto intraluminal vesicles (ILVs) of MVBs that subsequently fuse with lysosomes for degradation. We found that, in contrast to EGF stimulation, UVC or cisplatin exposure did not trigger EGFR ubiquitination (Fig. 4a). Ultraviolet exposure may downregulate the cellular ubiquitination machinery^25^, but EGF-dependent EGFR ubiquitination was still present, although slightly reduced, in UVC-pre-treated cells, showing that the ubiquitination capacity was only marginally abridged (Fig. 4b). Consistent with the absence of a role for ubiquitination in stress-induced EGFR trafficking, ubiquitination-deficient mutant EGFR-15KR^26^ accumulated on both MVB-limiting membranes and ILVs following UVC exposure (Fig. 4c), and recycled to the plasma membrane following p38 inhibition (Fig. 4d), just as control EGFR-wt, when expressed in porcine aortic endothelial (PAE) cells. Moreover, EGFR-15KR co-segregated with transfected OA1 following both EGF and UVC exposure, whereas, as reported above in HeLa and flox/flox MEFs, EGFR-wt only co-localized with OA1 following UVC exposure (Fig. 4e), suggesting receptor ubiquitination as critical for its retention on the degradative pathway.

**Figure 5:**
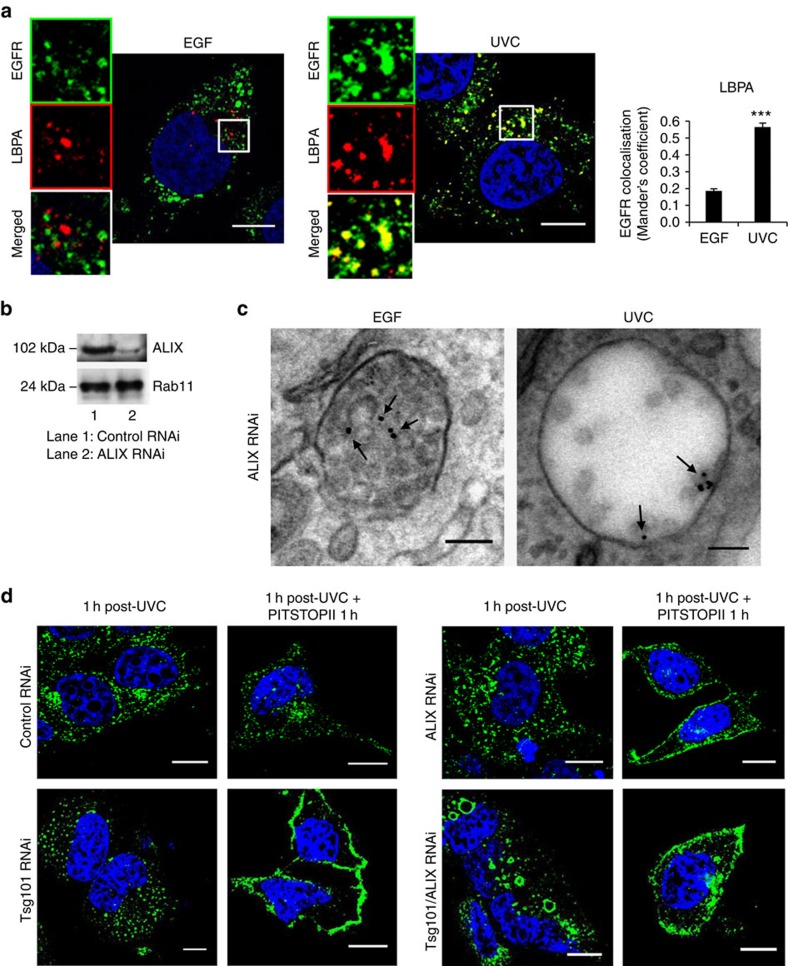
ALIX is required for sorting into ILVs and retention in MVBs of stress-internalized, but not EGF-bound EGFR. (**a**, left) Co-staining of EGFR (green) with LBPA (red) showed little co-localization in serum-starved HeLa cells treated with EGF for 30 min, but considerable overlap in cells 1 h after UVC exposure. Note that, in serum-free conditions, LBPA does not accumulate in lysosomes, facilitating the detection of MVB-specific labelling. (**a**, right) Quantification of co-localization of EGFR with LBPA in EGF-treated versus UVC-exposed serum-starved HeLa cells. Data are mean±s.e.m., ***P<0.001 (Student's *t*-test). (**b**) Lysates from HeLa cells transfected with control or ALIX siRNA were immunoblotted after 72 h for ALIX and Rab11 (as a loading control) to assess knockdown efficiency. (**c**) ALIX siRNA-treated cells were stimulated with EGF for 30 min, or exposed to UVC and incubated for 1 h, in the presence of anti-EGFR-gold, before EM processing. Ultrathin sections show gold (arrows) on ILVs of densely packed MVBs after EGF stimulation, but mainly on the limiting membrane of enlarged MVBs containing few ILVs in UVC-exposed cells. (**d**) Control, Tsg101, ALIX or Tgs101+ALIX siRNA-treated HeLa cells were exposed to UVC, incubated for 1 h and fixed, or further treated with PITSTOPII for 1 h. Immunostaining for EGFR (green) shows that depletion of Tsg101 or ALIX inhibits perinuclear EGFR accumulation, whereas EGFR redistributes to the plasma membrane upon PITSTOPII treatment, indicating that Tsg101 and ALIX are required for intracellular retention of EGFR. EGFR is found in very large vacuoles in double Tsg101+ALIX knocked-down cells after UVC exposure, before redistribution to the plasma membrane upon PITSTOPII treatment. Scale bars, 10 μm for confocal and 100 nm for EM pictures; 4,6-diamidino-2-phenylindole (DAPI)-stained nuclei, blue.

PMEL, which co-segregates with stress-activated EGFR, also undergoes ubiquitin-independent sorting onto ILVs, but independently of ESCRTs and requiring the tetraspanin CD63 (ref. 15). However, surprisingly, and despite lack of EGFR ubiquitination, knockdown of the ESCRT-0 component, Hrs, or the ESCRT-I component Tsg101 (ref. 24) resulted in inhibition of ILV formation and a reduced density of UVC-exposed EGFR on MVBs, where it predominantly localized to the limiting membranes (Fig. 4f, quantified in Supplementary Fig. 4a and validated with alternative siRNA sequences in Supplementary Fig. 4b). Presumably, for stress-internalized EGFR, the ESCRT-0 complex enables membrane recruitment of ESCRT-I rather than ubiquitinated cargo recognition^24^. Recent reports have shown that binding to any ESCRT can mediate ubiquitin-independent cargo sorting^27^, and the existence of a ESCRT-dependent, ubiquitin-independent pathway of ILV cargo sorting that depends on the ESCRT adaptor ALIX^28^,^29^. However, no role for ALIX has been demonstrated in sorting of EGF-stimulated EGFR onto ILVs or its targeting to lysosomes for degradation^30^. ALIX is recruited to MVBs by binding to the rare lipid lyso-bisphosphatidic acid (LBPA)^30^, which we previously showed to be absent from EGF-stimulated EGFR-containing MVBs^11^, but present in MVBs containing OA1 (ref. 16). Here we found extensive co-localization of stress-internalized, but not EGF-stimulated EGFR with LBPA in serum-starved cells (Fig. 5a). Consistently, ALIX knockdown in UVC-exposed cells resulted in a similar phenotype to that of Hrs and Tsg101 knockdown, in that UVC-exposed EGFR localized predominantly to the limiting membrane of MVBs containing fewer ILVs and reduced overall EGFR density (Fig 5b,c, quantified in Supplementary Fig. 4a and validated with an alternative siRNA sequence in Supplementary Fig. 4c). In contrast, ALIX depletion had no clear effect on MVBs containing EGF-stimulated EGFR. This is the first demonstration that ALIX is required for sorting of stress-internalized EGFR onto ILVs, but, in agreement with previous studies, ALIX is dispensable for sorting of ubiquitinated EGF-bound EGFR onto ILVs^30^.

The perinuclear accumulation of stress-internalized EGFR was remarkably stable and detected ≥4 h post UVC exposure (Supplementary Fig. 3d); however, ALIX or Tsg101 knockdown inhibited accumulation of UVC-stimulated EGFR in the perinuclear compartment (Fig. 5d). This was due to increased EGFR recycling because blockade of clathrin-dependent endocytosis after UVC-induced EGFR internalization (with PITSTOPII) caused the intracellular EGFR pool to return to the plasma membrane in ALIX- and/or Tsg101-depleted, but not control cells. Double Tsg101/ALIX knockdown resulted in accumulation of abnormally large endosomes with stress-internalized EGFR along their limiting membrane (Fig. 5d), suggesting cooperation between these two factors in stress-internalized EGFR sorting to ILVs. Thus, the intracellular sequestration of stress-internalized EGFR relies on the ESCRT/ALIX-dependent sorting of EGFR onto ILVs of perinuclear MVBs.

### Role of stress-induced EGFR traffic in regulating signalling

**Figure 6:**
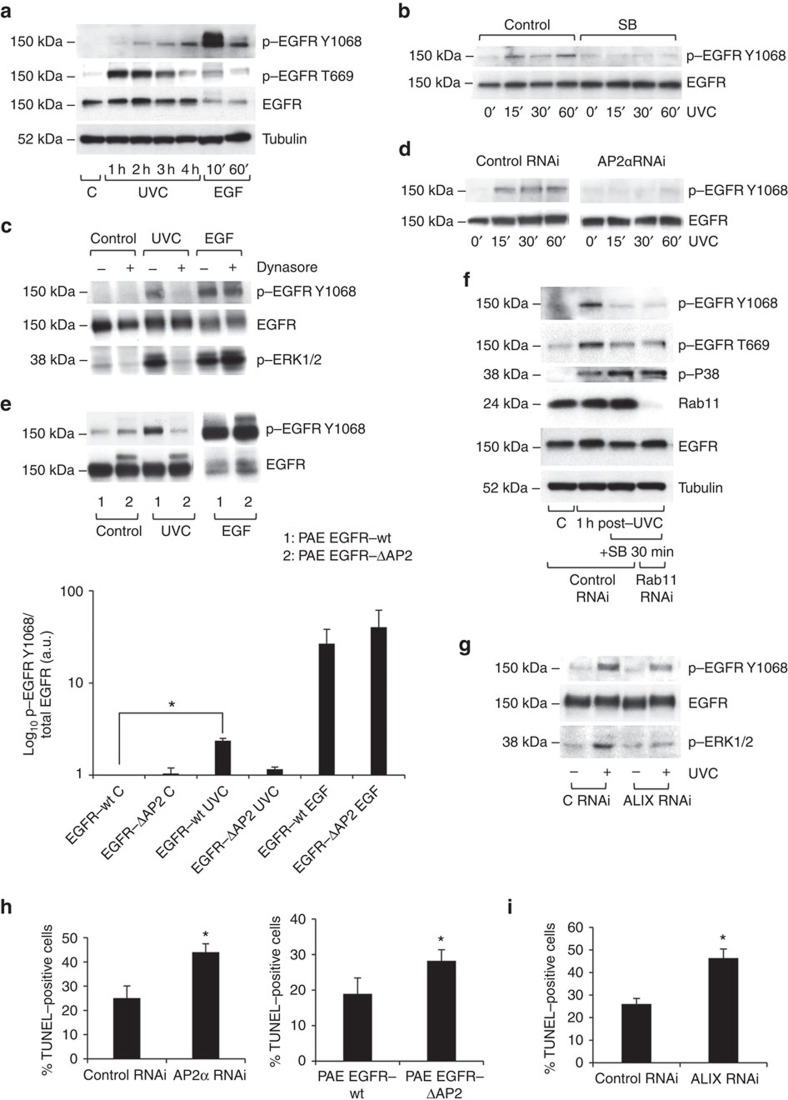
EGFR internalization and intracellular retention in a specific subset of MVBs is required for EGFR TK activation and delays onset of stress-induced apoptosis. (**a**) Immunoblotting HeLa lysates showed transient, strong EGFR-T669 phosphorylation and gradually increased EGFR-Y1068 phosphorylation after UVC exposure, compared with weak T669 and rapid, strong Y1068 signal after EGF stimulation. (**b**) Immunoblotting HeLa lysates pre-incubated for 30 min with SB, exposed to UVC and incubated in the continuous presence of SB, showed that EGFR-Y1068 phosphorylation requires p38 activity. (**c**) Immunoblotting HeLa lysates showed that 30 min pre-incubation with dynasore prevented EGFR-Y1068 and ERK1/2 phosphorylation induced 1 h post UVC exposure, but not that induced by 30 min exposure to EGF. (**d**) Immunoblotting HeLa lysates showed that AP2α siRNA treatment inhibited EGFR-Y1068 phosphorylation up to 1 h post UVC exposure. (**e**, top) Immunoblotting PAE lysates showed that EGF-stimulated Y1068-phosphorylation is similar in PAE EGFR-ΔAP2 and -wt cells. However, although EGFR-wt showed increased Y1068-phosphorylation 15 min post UVC exposure, −ΔAP2 did not. Note that exposure time for p-EGFR Y1068 in EGF-stimulated samples has been reduced to avoid film saturation. (**e**, bottom) Quantification of EGFR-Y1068 phosphorylation from above. Data were normalized to control (untreated) EGFR-wt and are mean±s.e.m. of three independent experiments, *P<0.05 (Student's *t*-test). (**f**) Immunoblotting HeLa lysates showed that 30 min SB treatment after UVC-induced EGFR internalization reduced EGFR-Y1068 phosphorylation that was not rescued by Rab11 siRNA treatment. (**g**) Immunoblotting HeLa lysates showed that ALIX siRNA treatment reduced EGFR-Y1068 phosphorylation and prevented ERK1/2 phosphorylation 1 h post UVC exposure. (**h**, left) AP2α RNAi results in increased percentage of TUNEL-positive HeLa cells 2 h post UVC exposure compared with control RNAi. (**h**, right) PAE EGFR-ΔAP2 showed increased TUNEL-positive cells 8 h post UVC exposure compared with EGFR-wt cells. Data are mean±s.e.m. of three independent experiments, *P<0.05 (Student's *t*-test). (**i**) ALIX RNAi causes a similar increase compared with control RNAi to that caused by AP2α RNAi in the percentage of TUNEL-positive HeLa cells 2 h post UVC exposure. Data are mean±s.e.m. of three independent experiments, *P<0.05 (Student's *t*-test).

What is the functional consequence of the p38-, ESCRT- and ALIX-dependent sequestration of stress-exposed EGFR in this perinuclear subpopulation of MVBs? A previous study has suggested little EGFR activation and increased susceptibility to apoptosis following stress-induced EGFR internalization^6^, whereas others have reported opposing results^31^. We found that stress-induced EGFR internalization, which correlated with p38-specific EGFR-T669 phosphorylation previously shown to be a requisite for internalization^31^, triggered gradual EGFR TK activation, as shown by EGFR-Y1068 phosphorylation (Fig. 6a). EGFR TK activity was not required for its internalization, as cells pre-treated with the EGFR inhibitor gefitinib, which prevented UVC-induced EGFR-Y1068 phosphorylation (Supplementary Fig. 5a), still displayed receptor internalization following UVC, but not EGF, exposure (Supplementary Fig. 5b, quantified in Supplementary Fig. 5c). The gradual activation of stress-induced EGFR contrasted with the rapid, strong EGFR TK activity triggered by EGF binding, a situation that accounts for the above-mentioned difference in ubiquitination, known to require a threshold of receptor tyrosine phosphorylation^32^. Anisomycin-induced p38 activation was sufficient to trigger EGFR internalization and TK activity (Supplementary Fig. 5d). Conversely, p38 inhibition abrogated UVC-induced EGFR TK activity (Fig. 6b). To directly test the role of EGFR internalization in its activation after stress exposure, endocytosis was inhibited by dynamin inhibition (Fig. 6c and Supplementary Fig. 5f) or AP2α RNA interference (RNAi; Fig. 6d), as AP2α has previously been shown^33^, and confirmed in the present study (Supplementary Fig. 5g), to be required for UVC/cisplatin-dependent EGFR internalization. Both treatments abolished stress-induced EGFR activation. Conversely, dynamin inhibition did not affect EGF-stimulated EGFR activity or ERK1/2 phosphorylation, whereas the latter was abolished by dynasore in UVC-exposed cells (Fig. 6c). The specificity for EGFR internalization versus other endocytosed cargoes was confirmed in PAE cells expressing EGFR-wt versus EGFR-ΔAP2, an EGFR mutant for AP2 binding^34^ that failed to undergo UVC-induced internalization (Supplementary Fig. 5h). We detected increased EGFR activity in EGFR-wt but not -ΔAP2 cells upon UVC exposure, whereas EGF triggered similar EGFR activation in EGFR-ΔAP2 and -wt cells (Fig. 6e). Thus, we show for the first time that although EGF induces EGFR TK activation on the plasma membrane^1^, stress-induced EGFR TK activation/signalling require its internalization.

In parallel with the dual role of p38 in stress-induced EGFR trafficking, p38 activity, present for ≥4 h following UVC exposure (Supplementary Fig. 5e), was required not only for EGFR activation linked to its internalization, but also for the maintenance of receptor activity post-internalization. Inhibiting p38 activity after UVC-induced EGFR activation and internalization caused EGFR-Y1068 phosphorylation to be lost (Fig. 6f). Continued EGFR activation does not appear to require direct p38-phosphorylation of EGFR at T669, as this was gradually lost following EGFR internalization (Fig. 6a). Loss of EGFR-Y1068 phosphorylation by p38 inhibition post-EGFR internalization was not rescued by inhibition of plasma membrane recycling with Rab11 RNAi, indicating that EGFR accumulation in recycling endosomes (Fig. 1d) is not sufficient to maintain EGFR TK activity (Fig. 6f). Conversely, specific retention of stress-internalized EGFR in MVBs is required to enable EGFR signalling, as knockdown of ALIX, which results in increased levels of EGFR plasma membrane recycling (Fig. 5d), causes loss of ERK1/2 phosphorylation, and a reduction in EGFR-Y1068 phosphorylation (Fig. 6g). The residual EGFR-Y1068 phosphorylation detected after ALIX knockdown is presumably due to EGFR being subjected to continuous p38-driven cycles of re-internalization and re-phosphorylation, but prolonged retention in MVBs is required to enable effective downstream signalling.

Although some stress-internalized EGFR is sequestered from the cytosol on ILVs of MVBs, it retains the capacity for Rab11-dependent recycling following p38 inhibition, indicating that mechanisms of back-fusion of EGFR-containing ILVs with MVB-limiting membranes are likely to be in place to allow for downstream signalling from the receptor.

### Role of stress-induced EGFR traffic in regulating apoptosis

To determine whether internalization- and intracellular sequestration-dependent signalling from the EGFR modulates cell survival following stress exposure, the effects of manipulating stress-induced EGFR trafficking on entry into apoptosis were assessed by TdT-mediated dUTP nick end labelling (TUNEL) assay. Inhibition of EGFR internalization by AP2α knockdown increased apoptotic cell numbers following UVC or cisplatin treatment (Fig. 6h and Supplementary Fig. 5i). The specificity of this effect on EGFR and no other clathrin-dependent cargo was established by measuring the apoptotic response to UVC in EGFR-wt versus −ΔAP2 PAE cells (Fig. 6h). Although EGFR endocytosis delayed UVC-induced apoptosis, long-term survival remained poor (9.06±0.01% surviving 24 h after UVC, assessed by MTT assay). Retention of stress-internalized EGFR within MVBs was also required to delay the onset of stress-induced apoptosis, as shown by increased apoptosis of UVC-treated cells following ALIX knockdown (Fig. 6i). Overall, these results indicate that stress-induced EGFR trafficking and retention into a subset of non-degradative MVBs allows for a fraction of receptors to become activated and signal, resulting in delayed onset of stress-induced apoptosis. Furthermore, MVBs containing stress-induced EGFR might act as a reservoir pool for intact receptor, protecting it from degradation and potentially modulating subsequent signalling upon ligand exposure.

## Discussion

**Figure 7:**
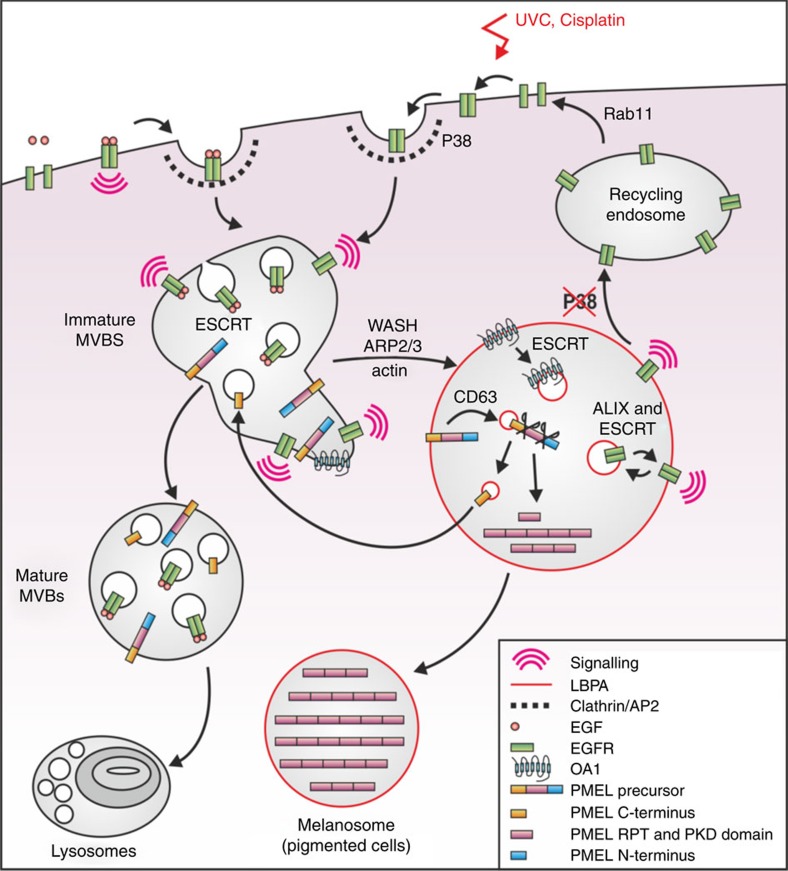
A working model for stress- versus EGF-induced EGFR trafficking. EGF stimulation triggers plasma membrane receptor activation and ubiquitination before internalization, whereas stress exposure induces p38-dependent EGFR-T669 phosphorylation and internalization into CCPs via interaction with AP2. EGF-bound and stress-internalized EGFR are then sorted from early endosomes onto separate MVB subsets, with stress-internalized EGFR undergoing WASH-dependent co-segregation with pre-melanosomal markers OA1 and fibrillar PMEL, whereas EGF-bound EGFR is retained in degradative MVBs by ubiquitin/ESCRT-dependent sorting onto ILVs and transported to lysosomes for degradation. Stress-exposed EGFR becomes activated post-internalization, and is largely retained in non-degradative MVBs from where it signals by the continued action of p38 in a mechanism that involves ALIX- and ESCRT-dependent receptor sorting onto ILVs, and may include cycles of internalization and back-fusion of ILVs with MVB-limiting membranes.

This study has led to the elucidation of a novel mechanism of stress-induced EGFR activation linked to its intracellular sequestration in a specific subset of non-degradative MVBs (see Fig. 7 for a schematic diagram). P38- and AP2-dependent EGFR internalization is followed by WASH-dependent segregation into a subset of MVBs that are distinct from those harbouring ligand-stimulated EGFR, but related to those that mature into melanosomes in pigmented cells. Although multiple populations of MVBs and ILVs have previously been identified, the mechanisms that regulate sorting between MVBs and their cargo with different destinations remain obscure. This study suggests that WASH-dependent regulation of actin dynamics may play an important role in this sorting.

The subset of MVBs that harbour stress-exposed EGFR has a number of novel features. In contrast to ligand-stimulated EGFR, sorting of EGFR onto the ILVs of these MVBs is independent of ubiquitination, but depends on the ESCRT machinery and ALIX. These ILVs must have the capacity to back-fuse with the MVB-limiting membrane, as they can return to the cell surface upon p38 inhibition. Although back-fusion of ILVs has previously been described^35^, this is the first demonstration that EGFR-containing ILVs can back-fuse. Moreover, although signalling from internalized EGFR has previously been demonstrated, this is the first example to our knowledge where internalization of EGFR and its retention in MVBs is required for the activation and maintenance of activity of the receptor. The intracellular location of the EGFR potentially regulates the availability of EGFR-interacting partners, such as tyrosine phosphatases or the TK Src, known to phosphorylate EGFR increasing its TK activity^36^. ALIX, which sorts EGFR onto ILVs, is also required to maintain EGFR signalling. This at first appears paradoxical because sorting onto ILVs sequesters the catalytic domain of the EGFR from the cytoplasm, and so would be expected to attenuate signalling. However, the demonstration that back-fusion can occur in these MVBs suggests a dynamic cycling between the limiting membrane and ILVs that together maintain the MVB location while also allowing signalling. This possibility is especially appealing given the localization of stress-internalized EGFR to LBPA-containing MVBs, and the previous demonstration of a role for LBPA and ALIX in back-fusion of ILVs within MVBs^30^. Elucidation of the role of ESCRTs/ALIX and p38 in combination in regulating the dynamic behaviour of the stress-exposed EGFR at the level of the MVB is a priority for future studies.

Although we primarily used UVC exposure to map EGFR trafficking and signalling, key experiments indicated that exposure to the chemotherapeutic drug cisplatin elicited similar EGFR trafficking and signalling. This has important implications for cancer therapy, as it could explain how chemotherapeutic agents such as cisplatin affect EGFR signalling, contributing to chemotherapy resistance and making tumours refractory to antibody therapies targeting the extracellular domain of EGFR. Further understanding of the molecular regulation of stress-internalized EGFR sorting and its influence on receptor TK activation and downstream signalling will be critical for interpreting its manipulation to increase cancer cell susceptibility to chemotherapy.

## Methods

### Cell culture and transfection

HeLa cells and MEFs (flox/flox and WASHOUT^23^) were cultured in DMEM+GlutaMAX (Life Technologies) containing 10% fetal calf serum. PAE cells stably expressing EGFR-wt, Y^974^RAL and LL^1010/1011^ −ΔAP2 (ref. 34), or -15KR^26^ were cultured in Hams F-12 (Lonza) with 10% fetal calf serum. In all experiments, cells were serum-starved overnight (o/n) at 37 °C before exposure to 100 J m^−2^ UVC with a CL-1000 Ultraviolet Crosslinker (UVP) or treatment with the indicated drugs in serum-free culture medium.

Transient cDNA transfections were performed with Lipofectamine 2000 reagent (Life Technologies) following the manufacturer's guidelines, for 48 h. Human wt EGFR and EGFR-GFP^37^, Rab5-Q79L-DsRed^10^, OA1-myc^16^ and PMEL^38^ expression vectors have all been described previously.

Transfection with siRNA (purchased from Dharmacon) was performed with Lipofectamine RNAiMAX (Life Technologies), for 72 h. Target sequences for AP2α^33^, Hrs and Tsg101 (ref. 39) have been described previously. Rab11 and ALIX target sequences were the ON-TARGETplus SMART pool (Dharmacon), and as described previously for ALIX RNAi_2 (ref. 40). Control siRNA was the AllStars-negative control (Qiagen).

See Supplementary Table 1 for details of siRNA sources and sequences.

### Reagents and antibodies

Reagents were used at the following concentrations: EGF (100 ng ml^−1^), SB202190 (10 μM), dynasore (80 μM), bafilomycin (200 nM) and anisomycin (100 μM) were from Sigma-Aldrich; cisplatin (200 μM) was from Mayne Pharma; PITSTOPII (20 μM) was from Abcam; gefitinib (Iressa/ZD1839, 10 μM) was from Astra Zeneca; EGF-AlexaFluor 488 and 647 (200 ng ml^−1^), Transferrin-AlexaFluor 555 (25 μg ml^−1^) and LysoTracker Red were from Life Technologies; EGF-horseradish peroxidase (EGF-HRP) (100 ng ml^−1^) was made from biotinylated EGF and streptavidin-HRP (from Life Technologies).

Antibodies for western blotting used were as follows: anti-phospho-EGFR-Y1068, anti-phospho-EGFR-T669, anti-phospho-p38 and anti-phospho-ERK1/2 from Cell Signaling; sheep anti-EGFR from Fitzgerald; anti-AP2α (a gift from M.S. Robinson laboratory); anti-Rab11 from Life Technologies; anti-Hrs from Enzo Lifesciences; anti-Tsg101 from GeneTex; anti-ubiquitin from Santa Cruz; anti-γ-tubulin (a gift from K. Matter lab) and anti-ALIX from Covalab.

Antibodies for immunofluorescence and cryo-immunoEM used were as follows: anti-EGFR antibody against the extracellular domain of the receptor isolated from the mouse 108 hybridoma (American Type Culture Collection); anti-Lamp1 (Abcam); anti-EEA1 (Santa Cruz); anti-Rab11 (Life Technologies); anti-LBPA (a gift from J. Gruenberg lab); rabbit anti-EGFR (Cell Signaling); anti-myc (Millipore); anti-PMEL fibrillar (HMB45 from Dako); anti-PMEL non-fibrillar (7E3 from Abcam); anti-PMEL Ct (αPEP13h, a gift from M.S. Marks lab); anti-GFP (Life Technologies) and sheep anti-EGFR (Fitzgerald).

See Supplementary Table 2 for details of antibody dilutions and catalogue numbers.

### Electron microscopy and cryo-immunoelectron microscopy

Colloidal gold sols (British Biocell International) were coupled to anti-EGFR 108 antibody by incubation with antibody at pH 9.3, followed by secondary stabilization with 1% BSA as described^41^. Antibody-gold conjugate was diluted in serum-free media containing 0.2% BSA and incubated with living cells. Conventional EM was performed as previously described^42^. Briefly, cells were cultured on Thermanox coverslips (Agar Scientific), fixed, processed, treated with tannic acid and mounted on Epon stubs. Epon was polymerized overnight at 60 °C, and the coverslips removed by heating. 70-nm sections were cut en face and stained with lead citrate before examination. 3,3′-Diaminobenzidine (DAB) reaction to reveal EGF-HRP was performed as in ref. 43 by incubation of cells in Tris-buffered saline containing 300–750 μg ml^−1^ DAB and 0.02% H_2_O_2_ for 30 min at 4 °C in the dark.

Cells were prepared for cryo-immunoEM by fixing with 4% paraformaldehyde+0.1% glutaraldehyde in 0.1 M phosphate buffer at pH 7.4 and pelleting in 12% gelatin. Subsequently, after infusion with 2.3 M sucrose, 80 nm sections were cut at −120 °C and collected in 2.3 M sucrose/2% methylcellulose (1:1 ratio). Sections were immuno-labelled as in ref. 41 by incubating with primary antibody and gold-tagged donkey anti-goat (Aurion) or gold-tagged protein A (UMC Utrecht).

Samples were viewed on a JEOL 1010 TEM, and images were acquired in a Gatan OriusSC100B charge-coupled device camera, and gold particles quantified in at least five cells per experiment using ImageJ.

### Immunofluorescence

Cells were fixed with 4% paraformaldehyde in PBS for 20 min, permeabilized in 0.5% Triton X-100 for 10 min and blocked with 5% BSA for 1 h. After labelling with primary antibodies o/n at 4 °C, cells were washed in PBS and incubated with Alexa Fluor-conjugated secondary antibodies for 45 min at room temperature. All antibody incubations were in 1% BSA in PBS.

For UVC+EGF sequential endocytosis experiments, HeLa cells were UVC-exposed and incubated for 1 h in the presence of excess anti-EGFR 108 antibody, followed by stimulation with EGF-AlexaFluor 488 for 30 min (or with EGF-AlexaFluor 647 for 3 h in Rab5-Q79L-DsRed-transfected cells), fixed and processed for immunofluorescence with AlexaFluor 555 secondary antibody to label EGFR. Note that 108 antibody labels all receptors present at the plasma membrane and does not significantly dissociate from EGFR following initial binding so that both non-EGF- and EGF-bound EGFR will be positive for 108.

Coverslips were mounted in Prolong Gold antifade reagent with 4,6-diamidino-2-phenylindole (Life Technologies) and images were acquired with a Leica DM-IRE2 microscope and TCS SP2 AOBS confocal system with a 63 × /1.4 numerical aperture oil-immersion objective (Leica). Quantification of co-localization was performed by measurement of Mander's coefficient in at least 20 cells per experiment using ImageJ.

### Immunoprecipitation and western blotting

EGFR was immunoprecipitated with 108 EGFR antibody bound to protein G Sepharose Fast Flow beads (Sigma). Immunoprecipitates were extensively washed and analysed by western blotting.

For western blotting, cells were lysed in lysis buffer [20 mM Tris, 150 mM NaCl, 1 mM EDTA, 1% NP40 (or Triton-X100), pH 7.4 plus protease inhibitor cocktail (Calbiochem set I) and phosphatase inhibitor cocktail (Calbiochem set II)]; lysates were fractioned by SDS–PAGE on 10% gels under reducing conditions and immunoblotted onto nitrocellulose membranes. Bands were detected by using enhanced chemiluminescence (Pierce) and exposed films developed on a SRX-101A Film Processor (Konica).

See Supplementary Figure 6 for uncropped scans of blots.

### Surface downregulation assay

Approximately 65,000 cells were seeded in six replicates per condition in a 48-well plate. Cells were serum starved o/n and following the appropriate treatment, fixed in 4% paraformaldehyde in PBS for 20 min. Half of the wells were permeabilized in 0.1% Triton X-100 for 8 min to quantify total EGFR level while the other half were left untreated to quantify surface level of EGFR. Cells were processed for in-cell western with 108 anti-EGFR antibody followed by IRDye 800CW donkey anti-mouse IgG secondary antibody (LI-COR) and DRAQ5 (to quantify cell number). Images were taken at 700 and 800 nm with a LI-COR Odyssey Infrared Imaging System and processed in ImageJ. Integrated density for the same area was quantified for each well and normalized by cell number.

### Neutral red assay

Approximately 20,000 cells were seeded in replicates of eight for each condition in a 96-well plate. Cells were serum-starved o/n and, following the appropriate treatment, incubated in 0.005% neutral red solution (Sigma) for 2.5 h to allow for crystal formation. The reaction was stopped in 50% ethanol+1% glacial acetic acid solution to dissolve the crystals. The content of each well was re-suspended, briefly spun and placed in a new 96-well plate. The absorbance at 550 nm was measured using a plate reader.

### TUNEL assay

Cells were fixed, permeabilized in 0.1% Triton X-100 for 5 min and processed using the *In Situ* Cell Death Detection Kit, TMR red (Roche) following the manufacturer's instructions. Five to ten images were taken in random areas and a minimum of 200 cells were counted in two replicates per experiment.

### MTT assay

Approximately 65,000 cells were seeded in replicates of eight for each condition in a 48-well plate. Cells were serum-starved o/n and, following the appropriate treatment, incubated in DMEM+0.5 mg ml^−1^ MTT reagent (3-(4,5-dimethylthiazol-2-yl)-2,5-diphenyltetrazolium bromide, Sigma) for 3 h to allow for crystal formation. Medium was removed and cells were incubated for 10 min in dimethylsulphoxide to dissolve the crystals, and absorbance at 570 nm was measured using a plate reader.

## Additional information

**How to cite this article**: Tomas, A. *et al*. WASH and Tsg101/ALIX-dependent diversion of stress-internalized EGFR from the canonical endocytic pathway. *Nat. Commun.* 6:7324 doi: 10.1038/ncomms8324 (2015).

## Supplementary Material

Supplementary Information Supplementary Figures 1-6, Supplementary Tables 1-2
