# Supplementary material for: WASH and Tsg101/ALIX-dependent diversion of stress-internalized EGFR from the canonical endocytic pathway
Source: Nat Commun. 2015 Jun 12;6:7324. doi: 10.1038/ncomms8324 (PMC4490399; doi:10.1038/ncomms8324)
Supplement: Supplementary Information — Supplementary Figures 1-6, Supplementary Tables 1-2 [file ncomms8324-s1.pdf]

Supplementary Figure 1

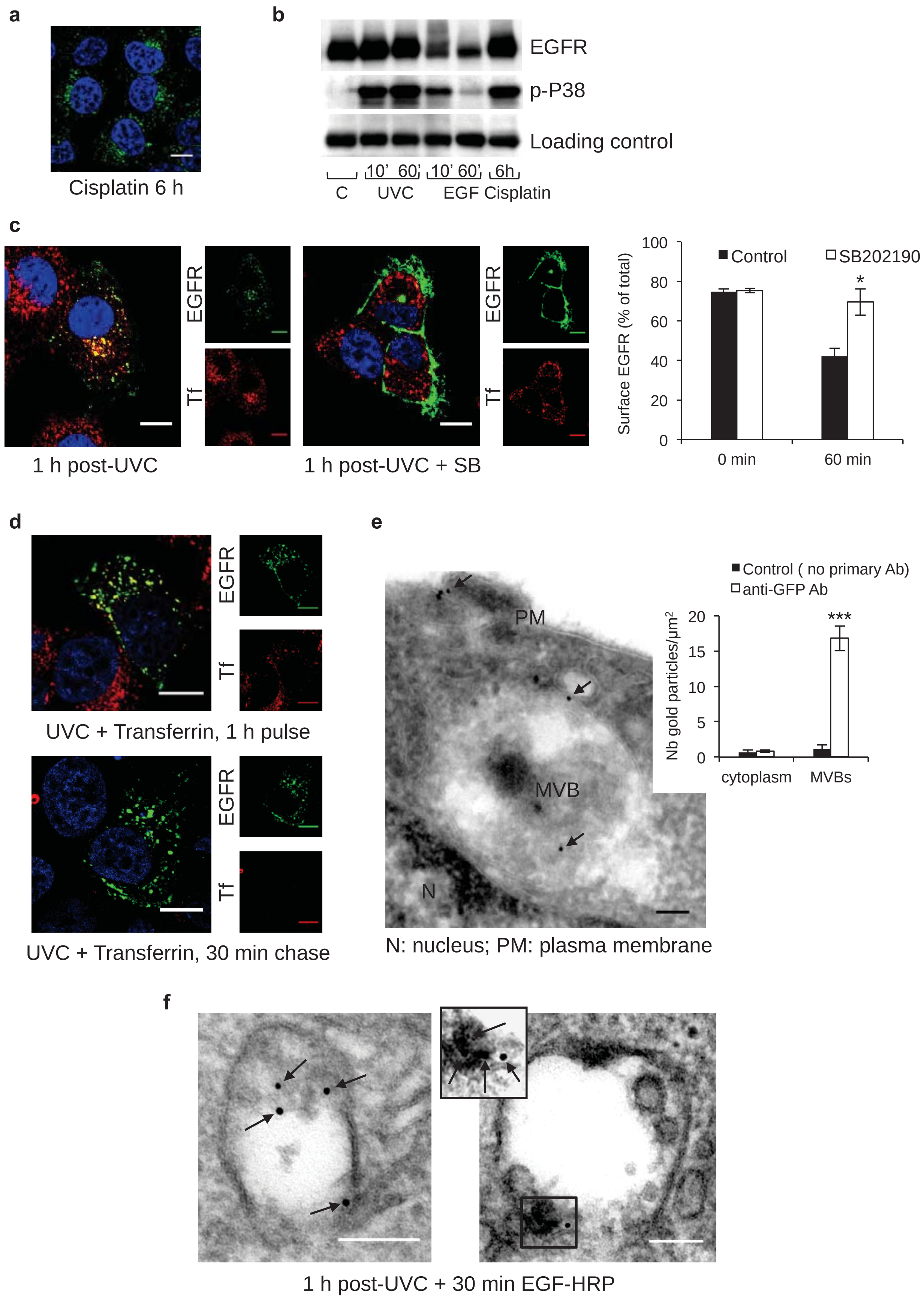

Supplementary Figure 1: **UVC- and cisplatin-induced internalisation and sequestration of EGFR, but not TfR, in a subset of perinuclear MVBs depends on p38 activity.** (**a**) Immunolocalisation of EGFR in HeLa cells treated with cisplatin for 6 h showing perinuclear accumulation, as for UVC. (**b**) Immunoblotted lysates from HeLa cells show strong p38 activation after UVC exposure or cisplatin treatment compared with much weaker transient activation after EGF stimulation. (**c**) EGFR, but not TfR, internalisation is p38-dependent: (**left**), HeLa cells transfected with EGFR-GFP (green) were pre-incubated for 30 min with or without the p38 inhibitor SB202190 (SB) before exposure to UVC and 1 h incubation with Transferrin-AlexaFluor 555 (Tf, red). Cells were fixed and analysed by confocal microscopy; (**right**), quantification of surface down-regulation in HeLa cells treated with or without SB before and 1 h after exposure to UVC. Data is mean ± s.e.m. of 3 independent experiments, **p* < 0.05 (Student's t-test). (**d**) Recycling of TfR is not affected by exposure to UVC: HeLa cells were transfected with EGFR-GFP (green) and exposed to UVC prior to 1 h pulse and 30 min chase with Transferrin-AlexaFluor 555 (Tf, red). Cells were fixed and analysed by confocal microscopy. (**e**) HeLa cells were transfected with EGFR-GFP, exposed to UVC and incubated for 1 h before being fixed and prepared for cryo-immunoEM. Ultrathin cryosections were labelled for GFP with 10-nm gold. EGFR-GFP (arrows) is present in the limiting membrane and ILVs of an MVB, as well as the plasma membrane. Inset graph shows quantification of EGFR-GFP density of gold-labelling per area of MVB vs. cytoplasm (excluding plasma membrane and nucleoplasm), in the presence or absence of primary anti-GFP antibody to show specificity of labelling. Data is mean ± s.e.m. of 3 independent experiments, ****p* < 0.001 (Student's t-test). (**f**) HeLa cells were exposed to UVC, incubated for 1 h with anti-EGFR-gold, washed and further incubated for 30 min with EGF-HRP before being fixed, developed with DAB reaction and processed for EM. Gold-labelled EGFR (arrows) localises to both HRP-negative (**left**) and HRP-positive (**right**) MVBs. Black box represents high-magnification area. Scale bars, 10 μm for confocal and 100 nm for EM images; DAPI-stained nuclei, blue.

Supplementary Figure 2

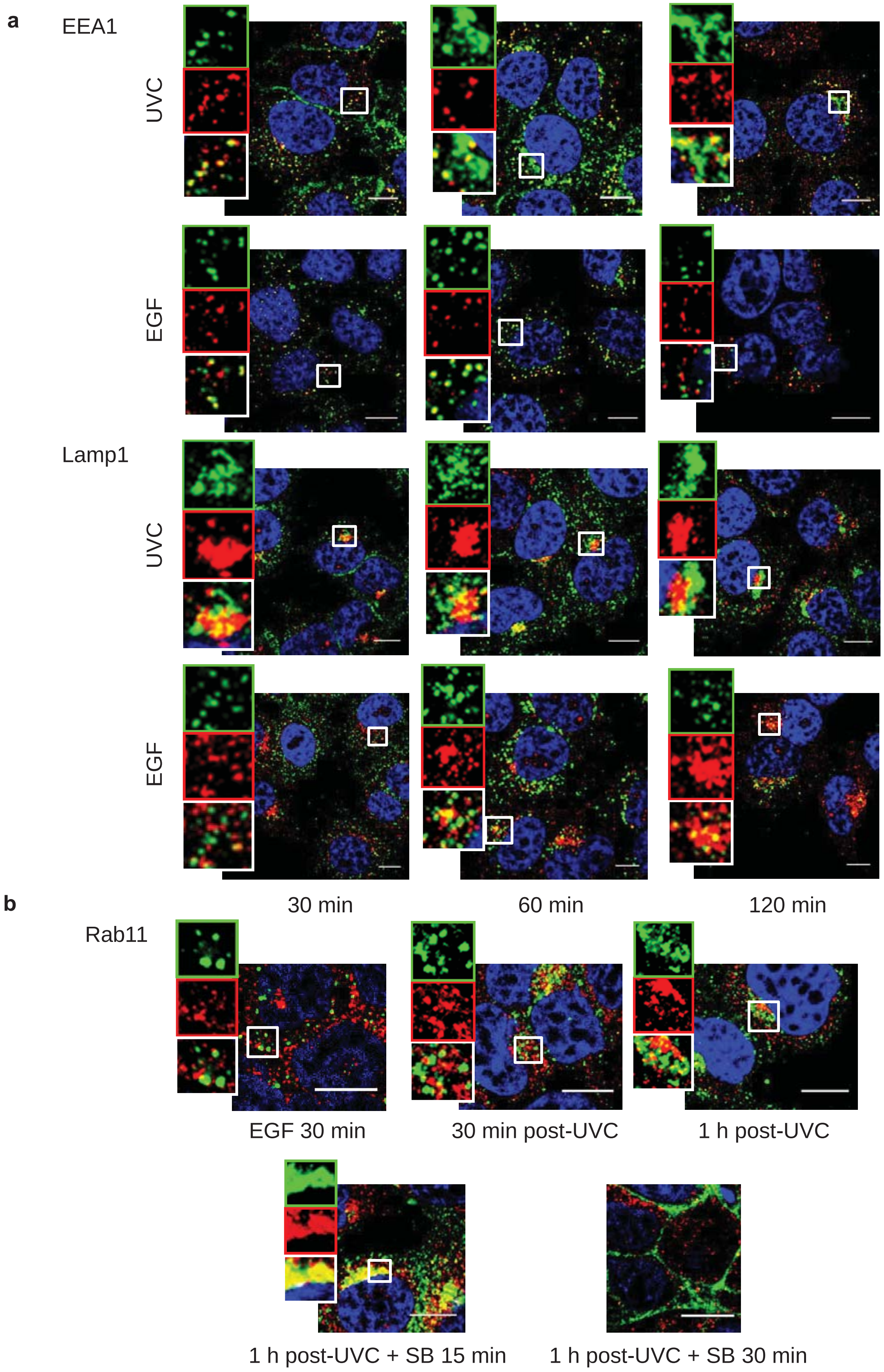

Supplementary Figure 2: **UVC-internalised EGFR passes through EEA1-positive early endosomes but then segregates from EEA1 and Lamp1, and upon p38 inhibition recycles via Rab11-positive recycling endosomes.** (**a**) HeLa cells were exposed to UVC or treated with EGF for the indicated times before fixation and processing for co-immunofluorescence of EGFR (green) and the early endosome marker EEA1 (red, **top** panels), or the lysosomal marker Lamp1 (red, **bottom** panels). Stress-internalised EGFR initially co-localises with EEA1 but this co-localisation is gradually lost as the receptor accumulates perinuclearly; however, unlike EGF-bound EGFR, stress-internalised EGFR does not co-localise with Lamp1 at later time points. White boxes represent high-magnification areas. (**b**) Co-immunolocalisation of EGFR (green) and Rab11 (red) in HeLa cells after treatment with EGF or exposure to UVC and subsequent treatment with SB. Note how EGFR and Rab11 only co-localise following 15 min of p38 inhibition, during EGFR recycling to the plasma membrane. White boxes represent high-magnification areas. Scale bars, 10 μm; DAPI-stained nuclei, blue.

Supplementary Figure 3

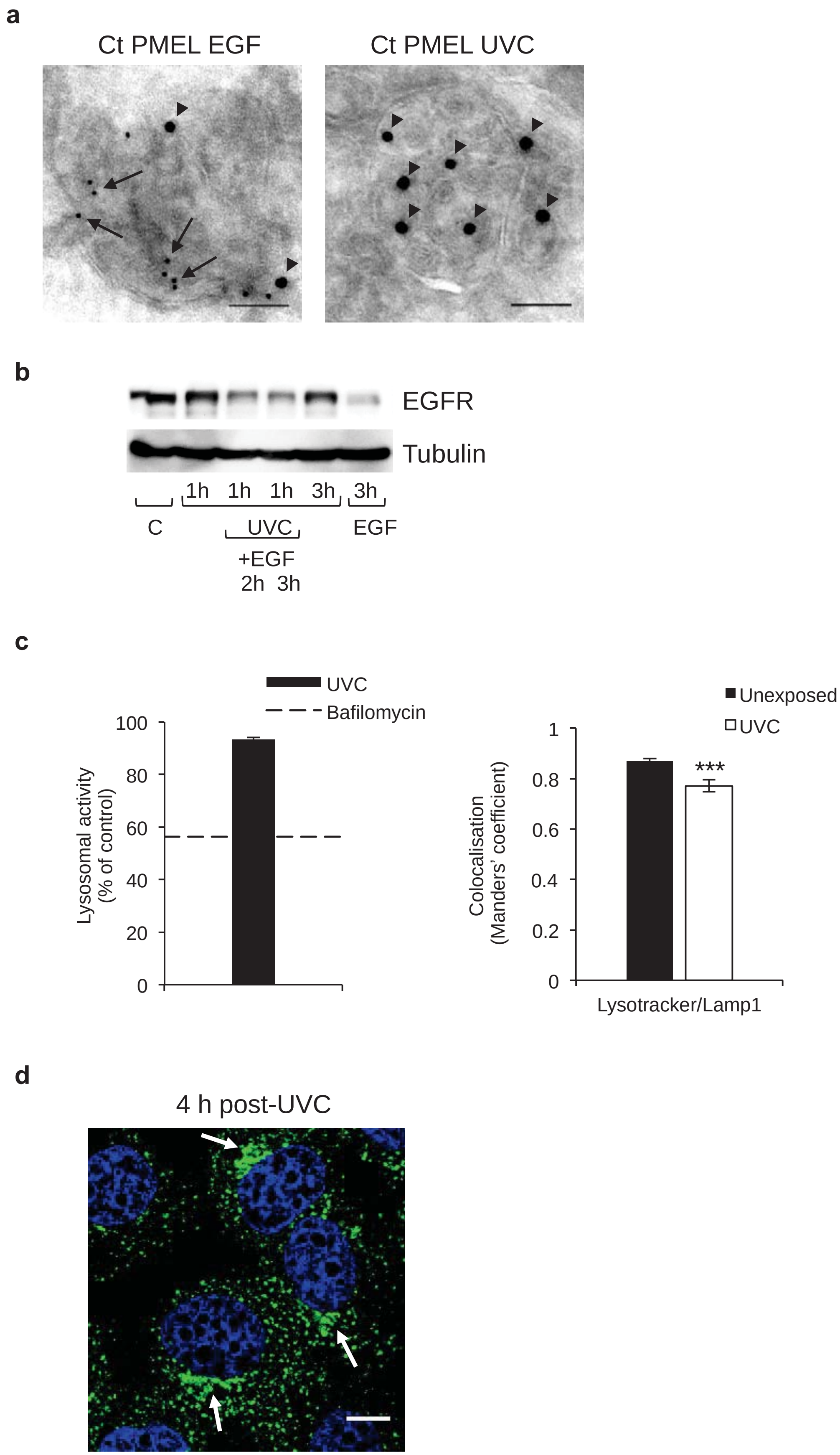

Supplementary Figure 3: **UVC-exposed EGFR is segregated in MVBs that do not fuse with lysosomes despite near-normal lysosome function.** (**a**) HeLa cells were transfected with PMEL, and either treated with EGF for 30 min in the presence of anti-EGFR-gold (arrows) or exposed to UVC and incubated for 1 h with anti-EGFR-gold before being fixed and prepared for cryo-immunoEM. Ultrathin cryosections were labelled for the C-terminal domain of PMEL with 15 nm-gold (arrowheads). C-terminal PMEL is found in the same MVBs as EGF-stimulated EGFR but is rarely found in MVBs containing UVC-internalised EGFR. (**b**) Western blot showing total EGFR and tubulin (as a loading control) in HeLa cells submitted to the indicated treatments. EGFR levels in cells exposed to UVC are similar to control untreated cells but are reduced in EGF-stimulated cells with or without prior exposure to UVC. (**c**) Lysosomal pH measured in HeLa cells 1 h after exposure to UVC by a neutral red assay (**left**), or by co-localisation of the acidic marker lysotracker with the lysosomal marker Lamp1 (**right**). Both experiments show a small decrease in lysosomal pH in cells exposed to UVC compared to the control. Dashed line indicates the reduction in lysosomal pH measured after 1 h incubation with the vacuolar-type H^+-ATPase inhibitor, bafilomycin. Data is mean ± s.e.m. of 3 independent experiments, ****p* < 0.001 (Student's t-test). (**d**) HeLa cells were exposed to UVC and incubated for 4 h before fixation. The intracellular localisation of EGFR (green) was analysed by immunofluorescence. Note the presence of EGFR in perinuclear clusters (arrows). Scale bars, 10 µm for confocal and 100 nm for EM images; DAPI-stained nuclei, blue.

Supplementary Figure 4

a

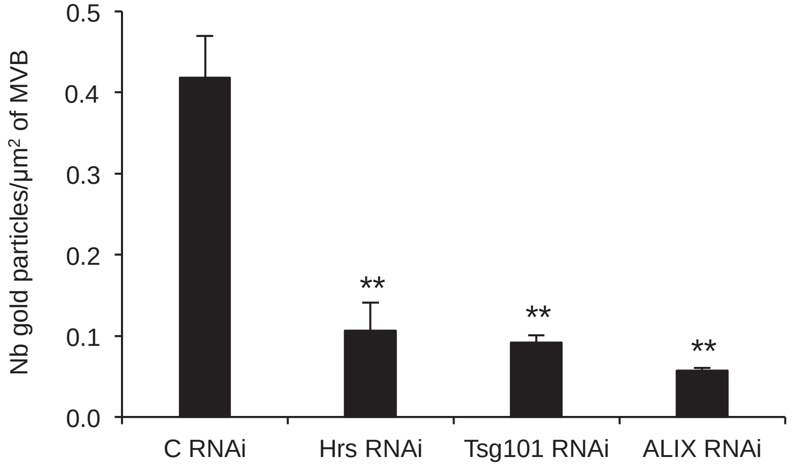

b

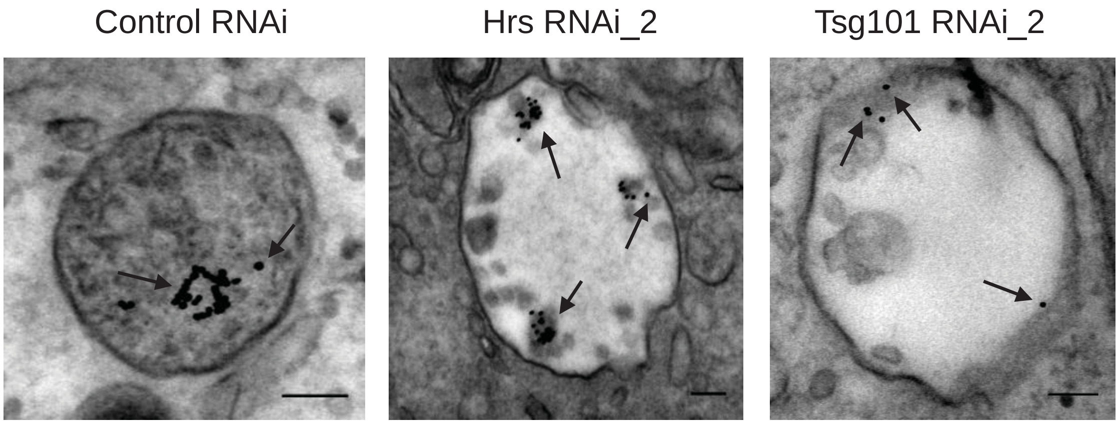

c

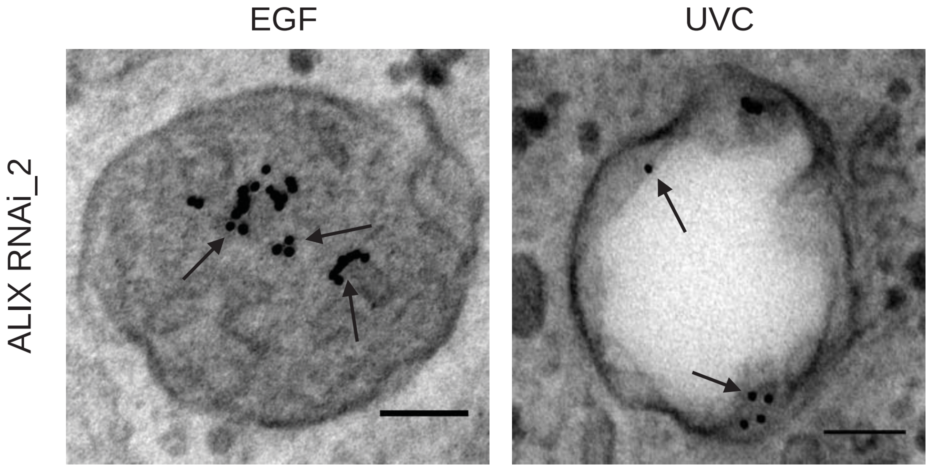

Supplementary Figure 4: **Quantification of the effects of ESCRT/ALIX depletion on UVC-exposed cells and verification with alternative siRNAs.** (**a**) Quantification of the density of EGFR-specific gold particles per area of MVB in electron micrographs from Control RNAi-, Hrs RNAi-, Tsg RNAi- and ALIX RNAi-treated HeLa cells 1 h post-UVC exposure. Data is mean ± s.e.m. of 3 independent experiments, $^{**}p < 0.01$ (Student's t-test). (**b**) HeLa cells were treated with alternative Hrs and Tsg101 siRNA sequences (Hrs RNAi_2 and Tsg101 RNAi_2), and processed as in **Fig. 4f** by exposing them to UVC followed by 1 h incubation with anti-EGFR-gold (arrows) prior to EM processing. Ultrathin sections show a similar phenotype consisting of enlarged MVBs containing reduced numbers of EGFR-positive ILVs in Hrs and Tsg101 siRNA-treated compared with control RNAi cells. (**c**) HeLa cells were treated with an alternative ALIX siRNA sequence (ALIX RNAi_2), and processed as in **Fig. 5c** by either 30 min EGF stimulation or 1 h post-UVC exposure in the presence of anti-EGFR-gold before EM processing. Ultrathin sections show a similar phenotype of gold (arrows) on ILVs of densely-packed MVBs after EGF stimulation, but mainly on the limiting membrane of enlarged MVBs containing few ILVs in UVC-exposed cells. Scale bars, 100 nm.

Supplementary Figure 5

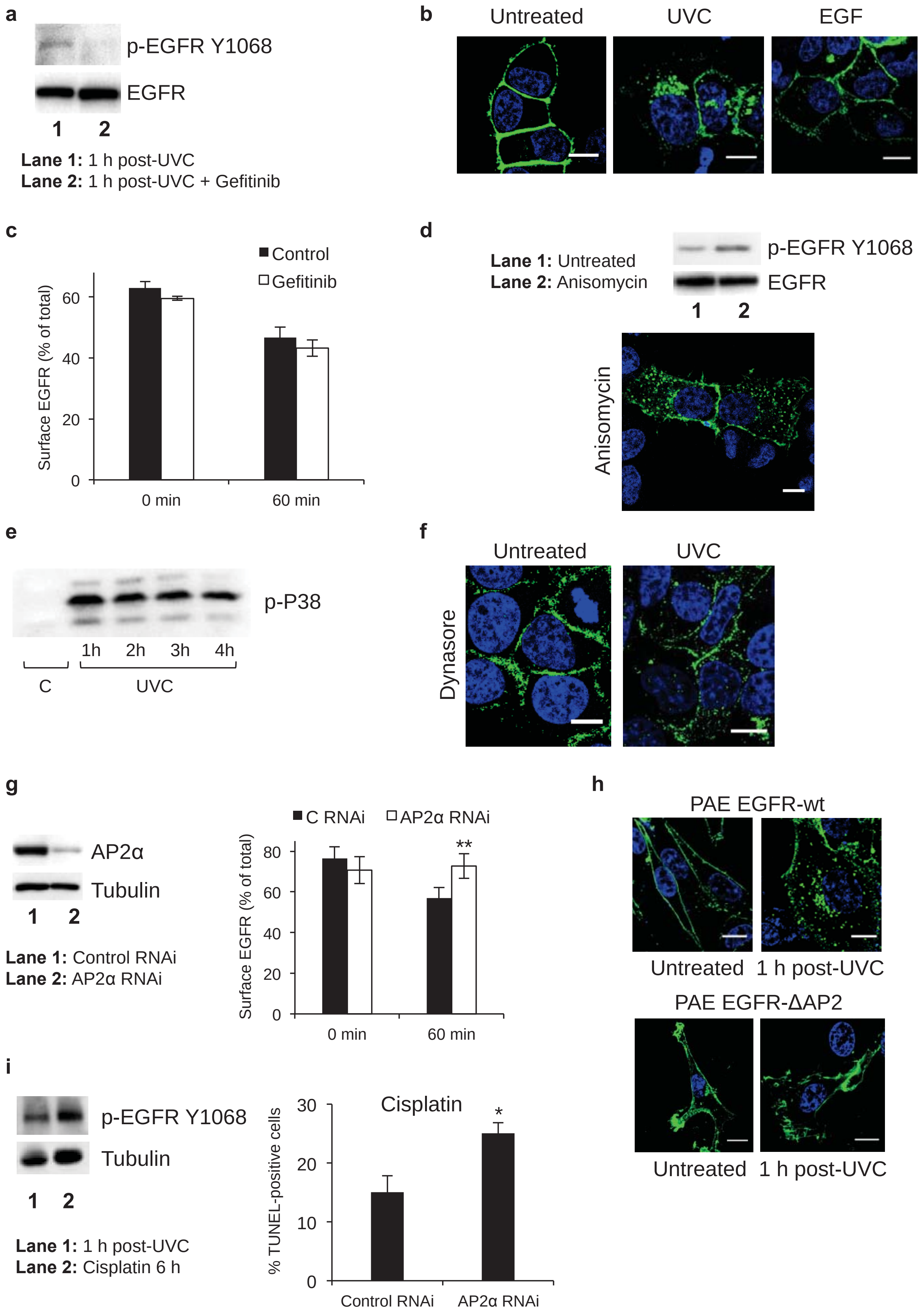

Supplementary Figure 5: **Regulation of UVC- and cisplatin-induced EGFR internalisation and effects of inhibition of EGFR internalisation on cisplatin-induced apoptosis.** (**a**) Immunoblotting HeLa lysates pre-incubated for 1 h with or without the EGFR TK inhibitor gefitinib and collected 1 h post-UVC exposure shows that gefitinib blocks UVC-stimulated EGFR-Y1068 phosphorylation. (**b**) Confocal microscopy of EGFR-GFP (green)-transfected gefitinib treated HeLa cells shows that EGFR TK inhibition prevents EGFR internalisation after 30 min EGF treatment, but does not affect perinuclear EGFR accumulation 1 h post-UVC exposure. (**c**) Quantification of EGFR surface down-regulation in HeLa cells treated or not with gefitinib before and 1 h post-UVC exposure. Data is mean ± s.e.m. (**d**, **top**) Immunoblotting HeLa lysates treated or not with the p38 activator anisomycin for 30 min shows anisomycin-dependent EGFR-Y1068 phosphorylation. (**d**, **bottom**) Confocal microscopy of EGFR-GFP (green)-transfected HeLa cells treated with anisomycin shows an intracellular pool of EGFR. (**e**) Immunoblotting HeLa lysates at the indicated times post-UVC exposure shows prolonged p38 activation. (**f**) HeLa cells were pre-incubated for 30 min with the dynamin inhibitor dynasore and exposed or not to UVC before fixation 1 h post-UVC exposure and immunofluorescence for EGFR (green). Dynamin inhibition greatly reduces stress-dependent EGFR internalisation. (**g**) HeLa lysates from control RNAi or RNAi specific for the α-subunit of AP2 (AP2α) were immunoblotted for AP2α and tubulin to assess knock-down efficiency (**left**), and EGFR surface down-regulation in control and AP2α RNAi-treated cells before and 1 h post-UVC exposure (**right**). AP2α knock-down suppresses UVC-induced EGFR surface down-regulation. Data is mean ± s.e.m. of 2 independent experiments, $^{**}p < 0.01$ (Student's t-test). (**h**) Immunolocalisation of EGFR (green) in stable PAE sublines expressing EGFR-wt or AP2 binding mutant (EGFR-ΔAP2) before and 1 h post-UVC exposure. PAE EGFR-wt but not -ΔAP2 undergo UVC-stimulated EGFR internalisation. (**i**, **left**) Western blot showing active EGFR and tubulin (as loading control) in HeLa lysates 1 h post-UVC exposure or after 6 h cisplatin treatment. (**i**, **right**) HeLa cells were transfected with control or AP2α siRNA, treated with cisplatin for 4 h and processed for TUNEL. The percentage of TUNEL-positive cells is increased in AP2α vs. control RNAi-treated cells. Data is shown as mean ± s.e.m. of 3 independent experiments, $^{*}p < 0.05$ (Student's t-test). Scale bars, 10 μm; DAPI-stained nuclei, blue.

Supplementary Figure 6

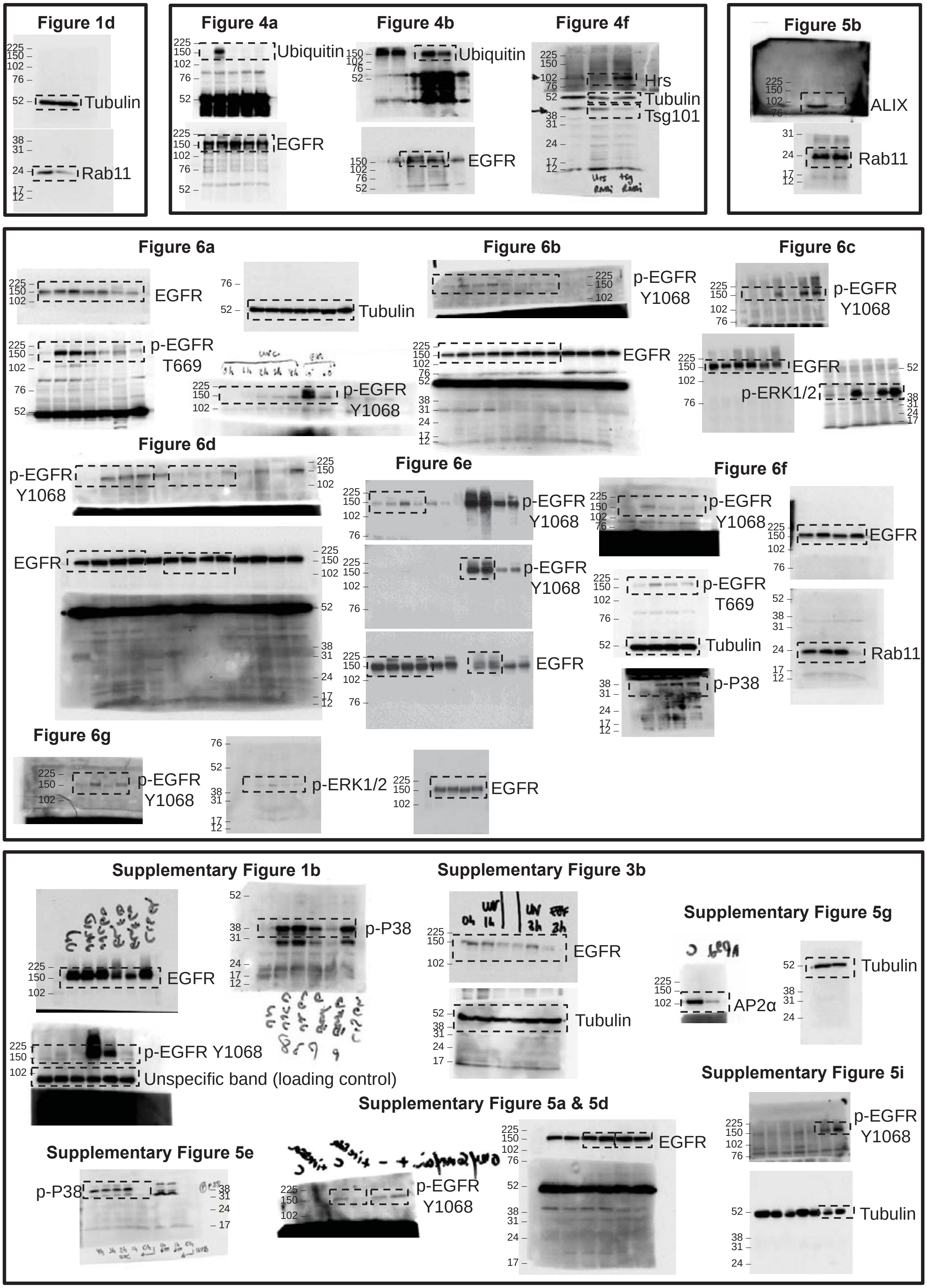

Supplementary Figure 6: Full scans of all blots presented in cropped form in figures in the primary and supplementary manuscript texts. Numbers represent molecular weight marker positions in kDa.

Supplementary Table 1: Sources and sequences of all the siRNAs used in this study.

Name	Reference or catalogue number	Target sequence
AP2α RNAi	Grandal et al., Traffic 2012	5'-GAGCAUGUGCACGCUGGCCA-3'
Rab11 RNAi	L-004726-00-0005 (Dharmacon)	ON-TARGETplus Human RAB11A siRNA SMARTpool
Hrs RNAi	Razi et al., Mol Biol Cell 2006	5'-AGAGACAAGUGGAGGUAAA-3'
Hrs RNAi_2	Razi et al., Mol Biol Cell 2006	5'-CGACAAGAACCCACACGUC-3'
Tsg101 RNAi	Razi et al., Mol Biol Cell 2006	5'-CCAGUCUUCUCUCGUCCUA-3'
Tsg101 RNAi_2	Razi et al., Mol Biol Cell 2006	5'-CCUCCAGUCUUCUCUCGUC-3'
ALIX RNAi	L-004233-00-0005 (Dharmacon)	ON-TARGETplus PDCD6IP siRNA SMARTpool
ALIX RNAi_2	Matsuo et al., Science 2004	5'-GCCGCUGGUGAAGUUCAUC-3'

Supplementary Table 2: Sources and dilutions of all the antibodies used in this study.

Name	Source and catalogue number	Dilutions
Anti-myc tag clone 4A6	05-724 (Millipore)	IF: 1/200<br>cryo-immunoEM: 1/50
Anti-PMEL fibrillar (HMB45)	M0634 (Dako)	IF: 1/200<br>cryo-immunoEM: 1/100
Anti-PMEL non-fibrillar (7E3)	ab117853 (Abcam)	IF: 1/200<br>cryo-immunoEM: 1/50
Anti-PMEL Ct (αPEP13h)	M.S. Marks lab, Pennsylvania	cryo-immunoEM: 1/25
Anti-Rab11	610657 (BD Biosciences)	IF: 1/80<br>WB: 1/1000
Anti-LAMP1 (H4A3)	ab25630 (Abcam)	IF: 1/400
Anti-EGFR	20-ES04 (Fitzgerald)	WB: 1/2000<br>cryo-immunoEM: 1/25
Anti-GFP	2555 (Life Technologies)	cryo-immunoEM: 1/50
Anti-phospho-p38	9211 (Cell Signaling)	WB: 1/500
Anti-phospho-ERK1/2	9106 (Cell Signaling)	WB: 1/1000
Anti-phospho-EGFR-T669	3056 (Cell Signaling)	WB: 1/500
Anti-phospho-EGFR-Y1068	2234 (Cell Signaling)	WB: 1/1000
Anti-EGFR	4267 (Cell Signaling)	IF: 1/50
Anti-AP2α	M.S. Robinson lab, Cambridge	WB: 1/500
Anti-EGFR (108)	Purified from HB-9764 (ATCC)	IF: 1/200
Anti-γ-tubulin	K. Matter lab, UCL	WB: 1/100
Anti-ALIX (PDCD6IP)	pab0204 (Covalab)	WB: 1/200
Anti-EEA1 (H-300)	sc-33585 (Santa Cruz)	IF: 1/50
Anti-Hrs (A-5)	ALX-804-382-C050 (Enzo)	WB: 1/2000
Anti-Tsg101 (4A10)	GTX70255 (GeneTex)	WB: 1/500
Anti-ubiquitin (P4D1)	sc-8017 (Santa Cruz)	WB: 1/200
Anti-LBPA	J. Gruenberg lab, Geneva	IF: 1/20
